# Outcomes of Percutaneous Revascularization in Severe Ischemic Left Ventricular Dysfunction

**DOI:** 10.1007/s11886-024-02045-2

**Published:** 2024-04-20

**Authors:** Roshan Bista, Mohamed Zghouzi, Manasa Jasti, Hady Lichaa, Jimmy Kerrigan, Elias Haddad, M. Chadi Alraies, Timir K. Paul

**Affiliations:** 1 University of Tennessee Health Science Center, Nashville, TN USA; 2 Ascension St., Thomas Hospital, Nashville, TN USA; 3 Detroit Medical Center, Cardiovascular Institute, Heart Hospital, Detroit, MI USA

**Keywords:** Percutaneous coronary intervention, Coronary artery disease, Optimal medical therapy, Left ventricular dysfunction, Myocardial viability

## Abstract

**Purpose of Review:**

This article presents a comprehensive review of coronary revascularization versus optimal medical therapy (OMT) in patients with severe ischemic left ventricular dysfunction.

**Recent Findings:**

The REVIVED-BCIS2 trial randomized 700 patients with extensive coronary artery disease and left ventricular (LV) ejection fraction (LVEF) ≤ 35% and viability in more than four dysfunctional myocardial segments to percutaneous coronary intervention (PCI) plus OMT versus OMT alone. Over a median duration of 41 months, there was no difference in the composite of all-cause mortality, heart failure hospitalization, or improvement in LVEF with PCI plus OMT versus OMT alone at 6 and 12 months, quality of life scores at 24 months, or fatal ventricular arrhythmia. The STICH randomized trial was conducted between 2002 and 2007, involving patients with LV dysfunction and coronary artery disease. The patients were assigned to either CABG plus medical therapy or medical therapy alone. At the 5-year follow-up, the trial showed that CABG plus medical therapy reduced cardiovascular disease-related deaths and hospitalizations but no reduction in all-cause mortality. However, a 10-year follow-up showed a significant decrease in all-cause mortality with CABG.

**Summary:**

The currently available evidence showed no apparent benefit of PCI in severe ischemic cardiomyopathy as compared to OMT, but that CABG improves outcomes in this patient population. The paucity of data on the advantages of PCI in this patient population underscores the critical need for optimization of medical therapy for better survival and quality of life until further evidence from RCTs is available.

## Introduction

Heart failure (HF) has emerged as a global health challenge, with its prevalence estimated to include 6.7 million individuals aged 20 years or older in the United States, and this number could surpass 8 million within the next decade [1, 2]. Coronary artery disease (CAD) is a significant cause of HF, as evidenced by a population-based study in Olmsted County revealing a threefold increase in HF risk among CAD patients [3]. Despite this established association, ischemic evaluation remains underutilized in patients presenting with acute onset HF, with only about a quarter undergoing such assessments during HF admissions [4].

The term “hibernating myocardium” describes viable myocardial tissue linked to left ventricular (LV) dysfunction stemming from chronically reduced coronary blood flow or ischemia [5]. This observation laid the groundwork for exploring the potential benefits of revascularization, contemplating that restoration of coronary blood flow could, in turn, partially or entirely reverse LV dysfunction [6, 7]. Surgical revascularization outcomes in this patient cohort have yielded mixed 10-year survival results [8, 9]. There is a paucity of data on percutaneous coronary intervention (PCI) outcomes, particularly outside of cardiogenic shock scenarios.

The debate surrounding the survival benefit of revascularization versus optimal medical therapy (OMT) in patients with severe LV dysfunction persists, particularly in the context of evolving and newer HF medical therapies. The Surgical Treatment for Ischemic Heart Failure (STICH) trial offered insights by demonstrating long-term (> 10 years) survival improvements in patients undergoing coronary artery bypass grafting surgery (CABG) combined with OMT, as opposed to OMT alone [8, 10, 11]. Notably, the benefits of revascularization, especially with PCI, remained largely unexplored in major PCI trials due to the exclusion of patients with severe LV dysfunction [12, 13].

The contemporary Revascularization for Ischemic Left Ventricular Dysfunction (REVIVED-BCIS2) trial emerges as the inaugural randomized controlled trial (RCT) scrutinizing the efficacy of PCI in conjunction with OMT for revascularization in patients with severe LV dysfunction [14••]. This article provides a comprehensive synthesis of the existing body of evidence on diverse revascularization modalities in patients with severe ischemic LV dysfunction, focusing on the recent REVIVED-BCIS2 trial on using PCI as a revascularization modality.

## Revascularization Studies in Patients with Severe Left Ventricular Dysfunction

The Coronary Artery Surgery Study (CASS) was a pivotal RCT conducted between 1975 and 1979, exploring the mortality benefits of CABG in stable ischemic heart disease versus medical therapy. The trial failed to unveil a significant mortality advantage among the groups, with consistent findings across subgroups based on the number of vessels affected, ejection fraction (EF), or a combination of these factors [15]. Intriguingly, a long-term survival analysis from the CASS registry paradoxically revealed poor survival in patients with two to three vessels disease and poor LV function under medical therapy, emphasizing the potential benefit of revascularization in this subgroup, primarily through CABG, though a small fraction underwent revascularization via PCI introduced in 1977 [16]. The STICH trial remains the singular study demonstrating robust long-term mortality benefits in patients with ischemic cardiomyopathy and severe LV dysfunction undergoing CABG, contributing to current practice guidelines favoring CABG over PCI [8]. A summary of the characteristics and results of these studies is provided in Table 1.

**Table 1:** Trials of CABG in ischemic LV dysfunction

Trials	Study design	Total patients	Control group	Intervention group	Primary outcome in the control group	Primary outcome in the intervention group	Conclusion
CASS (1983) [15]	Multicenter, RCT	780	Non-surgical (*n* = 390)	Surgical (*n* = 390)	1.4%, 1.2%, and 2.1% (annual mortality rate in single-, double-, and triple-vessel disease at 5-year follow-up) 1.6% (5-year average annual mortality rate) 51% (survival rates in patients with LVEF < 50% at 10-year follow-up)	0.7%, 1.0%, and 1.5%.(annual mortality rates in patients with single-, double-, and triple-vessel disease at 5-year follow-up) 1.1% (5-year average annual mortality rate) 79% (survival rates in patients with LVEF < 50% at 10-year follow-up)	The 10-year follow-up sub-group analysis showed significant mortality benefit in favor of CABG.
STICH (2011) [10]	Non-blinded, parallel-group, RCT	1212 (EF < 35% and CAD)	OMT alone (*n* = 602)	OMT plus CABG (*n* = 610)	41% (all-cause mortality) 33% (CV death)	36% (all-cause mortality) 28% (CV death)	5-year follow-up showed that CABG plus medical therapy reduced CV deaths and hospitalizations but did not reduce all-cause mortality The 10-year follow-up showed a significant decrease in all-cause mortality with CABG.

*LV* left ventricle, *CASS* Coronary Artery Surgery Study, *STICH* Surgical Treatment for Ischemic Heart Failure, *RCT* randomized controlled trial, *LVEF* left ventricular ejection fraction, *CABG* coronary-artery bypass graft, *EF* ejection fraction, *CAD* coronary artery disease, *OMT* optimal medical therapy, *CV* cardiovascular

With the continuous improvement in PCI technology since the late 1970s, there has been an escalating interest in utilizing PCI for revascularization in ischemic LV dysfunction. Despite being limited in number, retrospective studies have endorsed the feasibility and safety of PCI in severe LV dysfunction, proposing it as a potentially effective treatment, albeit with mixed results regarding mortality benefits in this patient population [17–21]. The Angina With Extremely Serious Operative Mortality Evaluation (AWESOME) study, a prospective RCT conducted between 1995 and 2000, compared PCI and CABG in patients with refractory angina deemed high risk for CABG, including those with LVEF < 35%. Results demonstrated comparable outcomes between CABG and PCI, with no difference in survival [22]. A meta-analysis of 19 studies involving 4766 patients with LVEF ≤ 40% undergoing PCI versus medical therapy suggested acceptable in-hospital and long-term mortality rates, comparable with outcomes seen in CABG patients [23]. The Heart Failure Revascularization Trial (HEART) randomly assigned patients with CAD, LVEF < 35%, and myocardial viability to either conservative treatment or coronary angiography with the intention to revascularize using PCI or CABG. There were 138 out of 800 initially planned patients enrolled, and out of 138 patients, 69 were assigned to coronary revascularization (either PCI or CABG). The study concluded that the conservative approach was just as effective as coronary angiography with an intention to perform coronary revascularization. However, the study was stopped early due to slow recruitment [24].

The REVIVED-BCIS2 was a multicenter, prospective, open-label RCT in the UK that randomized patients with LVEF ≤ 35% and extensive CAD defined as **B**ritish **C**ardiovascular **I**ntervention **S**ociety **j**eopardy **s**core (BCIS-js^3^ ≥ 6) to either PCI plus OMT vs. OMT alone. Attempted complete revascularization of all angiographically proximal stenoses supplying a viable area of myocardium was required in the study protocol [14••]. Complete revascularization was assessed utilizing the BCIS-js index, with 100% indicating complete revascularization [25]. The primary outcome was a composite end point of death from any cause or heart failure hospitalization over the minimum required follow-up of 2 years. The secondary major outcomes were EF assessed by echocardiography and quality of life assessed by the **K**ansas **C**ity **C**ardiomyopathy **Q**uestionnaire (KCCQ). A total of 700 patients were enrolled, and around 50% had a history of previous myocardial infarction (MI) in both groups, with previous revascularization with either PCI or CABG. Left main and three-vessel disease was reported in both groups at 13% and 40%, respectively. The reported anatomical revascularization index was 71% in the PCI group, and the median follow-up was 41 months. The primary composite outcome was not statistically different between the two groups, with an event rate of 37.2% vs. 38% in the PCI and OMT groups, respectively (HR 0.99; 95% CI 0.78–1.27). There was no statistically significant change in the EF between the two groups at 6 and 12 months, but the quality-of-life assessment showed significant improvement in the PCI group at 6 and 12 months compared to the OMT group. However, the quality-of-life assessment was similar at 24 months between the two groups. There is a concern about the study lacking granular details regarding the specific anatomy, severity, and location of the existing CAD and the lack of physiologic testing that could have guided the suitability for revascularization with OMT vs. OMT alone [26].

A recent subgroup analysis of the REVIVED-BCIS2 trial explored whether viability testing would identify patients for whom revascularization would be beneficial [27•]. The patients who underwent cardiovascular magnetic resonance (CMR) imaging and dobutamine stress echocardiography were included in this analysis. Wall motion was classified as either normal or dysfunctional. Furthermore, dysfunctional segments were further classified into viable or non-viable based on a standard definition of late 25% transmural gadolinium enhancement in the CMR imaging and contractile reserve identification on stress echocardiography [28, 29]. Surprisingly, there was no interaction between the extent of viability and the PCI effects on the primary composite outcome, which included all-cause death and heart failure hospitalization. Additionally, all secondary outcomes were no different in the PCI group compared to the OMT group. Conversely, the extent of non-viable myocardium was associated with increased primary and secondary outcome event rates [27•] (Table 2).

**Table 2:** Myocardial viability status/scar and outcomes in REVIVED-BCIS2 trial [27•]

Myocardium	Primary outcome: HR (95% CI)	All-cause death: HR 95% CI	CV death: HR 95% CI	HF hospitalization: HR 95% CI	Improved LVEF: HR 95% CI
Viable	0.98 (0.93–1.04)	0.98 (0.92–1.04)	0.97 (0.91–1.04)	0.96 (0.88–1.05)	1.01 (0.93–1.11)
Non-viable	1.07 (1.00–1.15)	1.10 (1.02–1.18)	1.13 (1.03–1.23)	1.04 (0.93–1.17)	0.82 (0.73–0.93)
Scar	1.18 (1.04–1.33)	1.21 (1.07–1.38)	1.28 (1.10–1.49)	1.11 (0.91–1.36)	0.69 (0.56–0.84)

*HR* hazard ratio, *CI* confidence interval, *CV* cardiovascular, *HF* heart failure, *LVEF *left ventricular ejection fraction

Primary outcome: composite of all-cause death or hospitalization for heart failure

## Mechanical Circulatory Support

The integration of mechanical circulatory support originated from its use in surgical revascularization patients [30]. Early observational studies hinted at the potential benefits of MCS in patients undergoing revascularization. Noteworthy evidence regarding revascularization in severe LV Systolic dysfunction with hemodynamic support emerged from the Balloon Pump–Assisted Coronary Intervention Study (BCIS-1) and the PROTECT II trials, comparing Impella 2.5 Left Ventricular Assist Device to the intra-aortic balloon pump (IABP) (Table 3) [31, 32]. These trials demonstrated that high-risk patients could undergo PCI without hemodynamic support, and either IABP or Impella 2.5 is acceptable if support is necessary. Similarly, participants in the PROTECT II trial and the cVAD (catheter-based ventricular assist device) registry, with severe LV dysfunction undergoing Impella or IABP-supported PCI, demonstrated increased LVEF [33]. Similar results were noted in a case series of 11 patients, in which patients with ischemic cardiomyopathy undergoing left ventricular assist device-supported high-risk PCI exhibited remarkable improvements in LVEF [34].

**Table 3:** Hemodynamic supported PCI in patients with severe LV dysfunction

Trials	Total patients	LVEF	Control group	Treatment group	Findings
BCIS-1 (2010) [31]	301	≤ 30%	No IABP (150)	IABP (151)	There was no significant difference in MACE or mortality at 28 days
PROTECT II (2012) [32]	452	≤ 30%	IABP (223)	Impella (225)	There is no difference in reducing adverse events at 30 or 90 days

*PCI* percutaneous coronary intervention, *LV* left ventricle, *BCIS* Balloon Pump–Assisted Coronary Intervention Study, *LVEF* left ventricular ejection fraction, *PROTECT II* prospective randomized clinical trial of hemodynamic support with Impella 2.5 vs. intra-aortic balloon pump in patients undergoing high-risk PCI, *IABP* intra-aortic balloon pump, *MACE* major adverse cardiac events

## Myocardial Viability and Revascularization in Patients with Ischemic Cardiomyopathy

A retrospective study and meta-analysis conducted in the past with the inclusion of 24 myocardial viability studies, including patients with stable CAD and left ventricular dysfunction, showed that those who exhibit myocardial viability on noninvasive tests have better chances of survival [35]. However, the PARR-2 trial (F-18-Fluorodeoxyglucose Positron Emission Tomography Imaging-Assisted Management of Patients With Severe Left Ventricular Dysfunction and Suspected Coronary Disease) did not demonstrate a significant reduction in cardiac events for patients with CAD and severe LV dysfunction who were being considered for revascularization or transplant when they were randomized to standard care versus FDG-PET-assisted management at 1-year and 5-year follow-up [36]. The results of the STICH trial in 2011 also showed that the presence of myocardial viability did not provide benefits for patients undergoing CABG compared to those receiving OMT alone after considering multiple variables (Table 4) [37]. In a subgroup study of the STICH trial, patients with CAD, severe LV dysfunction, and inducible ischemia (determined by either a radionuclide stress test or a dobutamine stress echocardiogram) were randomized to either medical therapy or CABG. There was no significant difference between the two groups regardless of the presence or absence of inducible ischemia [38]. A systematic review and meta-analysis published in 2015 that included 32 non-randomized and 4 randomized studies provided inconclusive evidence regarding the usefulness of myocardial viability testing as a deciding factor in the revascularization process for patients with CAD and LV dysfunction. With similar findings as STICH, the REVIVED-BCIS subgroup analysis showed a lack of benefit in assessing myocardial viability in guiding treatment decisions for this group of patients [39].

**Table 4:** Myocardial viability and survival in STICH trial [37]

Myocardial viability status	Probability of death in CABG group at 5 years	Probability of death in Medical Therapy Group at 5 years	HR (95% CI)	
Viable (total 487)	31.2% (*N*-83 deaths)	35.4% (*N*-95 deaths)	0.70 (0.41–1.18)	No significant difference in mortality in CABG vs. medical treatment
Non-viable (total 114)	41.5% (*N*-25 deaths)	55.8% (*N*-33 deaths)	0.86 (0.64–1.16)	No significant difference in mortality in CABG vs. medical treatment

*STICH* Surgical Treatment for Ischemic Heart Failure, *HR* hazard ratio, *CI* confidence interval, *N* number

## Optimal Medical Therapy

Guideline-directed medical therapy (GDMT) with beta-blockers, renin-angiotensin-aldosterone (RAAS) inhibitors [including angiotensin-converting enzyme inhibitors (ACEi), angiotensin receptor blockers (ARB), angiotensin receptor-neprilysin inhibitors (ARNi)], mineralocorticoid receptor antagonists (MRA), and sodium-glucose cotransporter 2 inhibitors (SGLT2i) remains pivotal in reducing the morbidity and mortality in patients with heart failure with reduced ejection fraction (HFrEF) [40, 41]. The 2022 American College of Cardiology (ACC)/American Heart Association (AHA) Heart Failure guideline recommendations regarding medical therapy for HFrEF to reduce morbidity and mortality are as follows [40].

1. ARNi in New York Heart Association (NYHA) class II–III symptoms (with use of an ACEi, or ARB if ARNI use “is not feasible”) are class 1-A recommendations.
2. Beta-blockers (metoprolol succinate, bisoprolol, or carvedilol) have Class 1-A recommendations.
3. MRA in patients with NYHA II-IV is a 1-A recommendation provided eGFR is > 30 mL/min/1.73 m^2^ and serum potassium is < 5 mEq/L.
4. SGLT2i is a Class 1-A recommendation irrespective of the presence of type 2 diabetes.
5. In patients who self-identify as African American with NYHA class III-IV who are receiving OMT, the addition of hydralazine and isosorbide dinitrate is a Class 1-A recommendation.

## Discussion

Coronary artery revascularization via CABG in severe LV dysfunction (LVEF < 35%) has been recommended in the 2021 ACC/AHA/SCAI guidelines for coronary artery revascularization [42] as well as the 2022 American College of Cardiology (ACC)/American Heart Association (AHA) Heart Failure guidelines [40]. This recommendation is mainly based on 10-year follow-up data from the STICH trial that showed a survival benefit with CABG compared to medical treatment. In appropriately selected patients, CABG remains a reasonable option for coronary revascularization, as REVIVED-BCIS2 failed to show benefit for those with severe ischemic cardiomyopathy who underwent PCI. However, we await long-term follow-up results of REVIVED-BCIS2 to fully understand the benefit (or lack thereof) of PCI in this group, especially for those who are not surgical candidates. Furthermore, we recommend the pursuit of future trials comparing PCI versus CABG in those with ischemic cardiomyopathy to help practitioners choose the best revascularization technique in this patient population. With the current evidence that PCI is not superior to OMT in severe ischemic cardiomyopathy, being aggressive in optimizing GDMT for HFrEF, including early initiation and rapid up-titration of GDMT to maximum tolerated doses while monitoring patient adherence, is essential.

Myocardial viability or inducible ischemia has been used to guide revascularization, but no convincing data supports this approach. The STICH viability and ischemia sub-group analysis and REVIVED-BCIS2 viability sub-group analyses have shown no benefit for patients when using myocardial viability testing or left ventricular inducible ischemia evaluation to guide management on coronary revascularization [27•, 37, 38]. Similarly, the PARR-2 Trial did not show a significant difference between inducible ischemia-guided management vs. standard care [43]. With the available data, myocardial viability or left ventricular ischemia testing is not compulsory to guide management in patients with CAD and severe left ventricular dysfunction. The heart team discussion is always beneficial in complex decision-making situations.

## Current Guidelines on Revascularization in Ischemic Cardiomyopathy

The 2021 ACC/AHA/SCAI guideline for coronary artery revascularization recommendations for patients with stable ischemic heart disease and multivessel CAD with anatomy suitable for either PCI or CABG are as follows [42].

If the patient’s LVEF is less than 50%, the first step is to determine whether CABG is suitable for the patient.

- If the patient is a CABG candidate and LVEF < 35%, then CABG is strongly recommended (Class 1 B-R).
- If LVEF is between 35 and 50%, then CABG is reasonable (Class 2a).
- If the patient is not suitable for CABG and LVEF is < 50%, then the recommended course of action is to discuss the case with a heart team and continue with GDMT, with or without PCI, depending on individual patient’s circumstances after shared decision making.

The 2018 ESC/EACTS Guidelines on myocardial revascularization give similar recommendations as the 2021 ACC/AHA/SCAI guideline for patients with CAD and severe left ventricular dysfunction [44].

## Conclusion

There is a lack of data on the benefit of PCI plus OMT versus OMT alone in patients with severe left ventricular dysfunction and multivessel CAD. Additionally, there is a paucity of data regarding CABG with OMT versus OMT in patients with severe ischemic cardiomyopathy. It is of the utmost important to optimize guideline directed medical therapy, including early initiation and rapid up-titration to maximum tolerated doses, and follow the current guideline recommendations on revascularization strategy until further evidence becomes available. Additionally, current available data does not support the routine use of myocardial viability assessment as a decision-making tool to guide coronary revascularization. Future RCTs are warranted comparing PCI versus CABG while on modern OMT in hopes of helping to guide revascularization strategies with the hope of improving patient outcomes.

## Data Availability

No datasets were generated or analyzed during the current study.
